# GRASP2: fast and memory-efficient gene-centric assembly and homolog search for metagenomic sequencing data

**DOI:** 10.1186/s12859-019-2818-1

**Published:** 2019-06-06

**Authors:** Cuncong Zhong, Youngik Yang, Shibu Yooseph

**Affiliations:** 1 Department of Electrical Engineering and Computer Science, University of Kansas, Lawrence, KS 66045 USA; 2 National Marine Biodiversity Institute of Korea, 101-75, Jangsan-ro, Janghang-eup, Seochun-gun, Chungchungnam-do 33662 South Korea; 3 Department of Computer Science, University of Central Florida, Orlando, FL 32816 USA

## Abstract

**Background:**

A crucial task in metagenomic analysis is to annotate the function and taxonomy of the sequencing reads generated from a microbiome sample. In general, the reads can either be assembled into contigs and searched against reference databases, or individually searched without assembly. The first approach may suffer from fragmentary and incomplete assembly, while the second is hampered by the reduced functional signal contained in the short reads. To tackle these issues, we have previously developed GRASP (Guided Reference-based Assembly of Short Peptides), which accepts a reference protein sequence as input and aims to assemble its homologs from a database containing fragmentary protein sequences. In addition to a gene-centric assembly tool, GRASP also serves as a homolog search tool when using the assembled protein sequences as templates to recruit reads. GRASP has significantly improved recall rate (60–80% vs. 30–40%) compared to other homolog search tools such as BLAST. However, GRASP is both time- and space-consuming. Subsequently, we developed GRASPx, which is 30X faster than GRASP. Here, we present a completely redesigned algorithm, GRASP2, for this computational problem.

**Results:**

GRASP2 utilizes Burrows-Wheeler Transformation (BWT) and FM-index to perform assembly graph generation, and reduces the search space by employing a fast ungapped alignment strategy as a filter. GRASP2 also explicitly generates candidate paths prior to alignment, which effectively uncouples the iterative access of the assembly graph and alignment matrix. This strategy makes the execution of the program more efficient under current computer architecture, and contributes to GRASP2’s speedup.

GRASP2 is 8-fold faster than GRASPx (and 250-fold faster than GRASP) and uses 8-fold less memory while maintaining the original high recall rate of GRASP. GRASP2 reaches ~ 80% recall rate compared to that of ~ 40% generated by BLAST, both at a high precision level (> 95%). With such a high performance, GRASP2 is only ~3X slower than BLASTP.

**Conclusion:**

GRASP2 is a high-performance gene-centric and homolog search tool with significant speedup compared to its predecessors, which makes GRASP2 a useful tool for metagenomics data analysis, GRASP2 is implemented in C++ and is freely available from http://www.sourceforge.net/projects/grasp2.

## Introduction

Metagenomics is a culture-independent approach for studying the genomic content of a given microbial community. In a typical metagenomics study, the DNA of the microbes from an environmental sample is extracted and sequenced using next-generation sequencing (NGS) technologies. Analysis of metagenomics data derived from medium- or high-complexity microbial communities is challenging due to the high taxonomic and genomic diversity of the constituent microbes, and also often due to insufficient and/or uneven sequencing coverage. De novo assembly (that aims at reconstructing individual microbial genomes from the sequencing reads) of such data sets is often incomplete and fragmentary, hampering downstream functional and taxonomic analysis. For example, the MetaHIT (Metagenomics of the Human Intestinal Tract) project data assembly resulted in a contig N50 of only 2.2 kb and left over 53% of the reads unassembled [1].

Assembly-independent analysis methods directly annotate individual reads by searching for them against available databases. These databases may contain fully sequenced genomes, proteins and protein domains, as well as marker genes with annotated taxonomy. Significant hits against the databases suggest homology between the reads and sequences in databases, allowing us to infer the function and taxonomy of the individual reads and subsequently predict the function of the entire community. However, this approach relies heavily on the completeness of the existing databases. In practice, such databases are rarely available, except for simple and well-studied communities, with microbial diversity in most environments not being sufficiently well-characterized or sequenced. In this case, most database searches involve moderate- or remote-homology detections, which are more challenging compared to close-homology detection. Such homolog searches are further confounded by the short length of the reads, which contain only a limited amount of functional and taxonomic signal.

We attempted to tackle this issue by overlapping the reads to make them longer and reconstruct the lost functional and taxonomic signal. The read-overlap detection is similar to those employed in de novo assembly. However, we did not perform excessive error correction and aggressive graph filtering and pruning as most assemblers do, which allows us to retain most polymorphisms from low-abundant genomes. Furthermore, we performed read overlap detection on the short-peptide sequences translated from the nucleotide reads (using FragGeneScan [2]); this approach has been shown to more effectively reconstruct protein sequences due to the collapse of synonymous mutations in the amino-acid space [3]. Based on such an intuition, we previously developed a simultaneous alignment and assembly algorithm called GRASP (Guided Reference-based Assembly of Short Peptides), that aims to find paths corresponding to homologs of a query/reference protein in the conceptual overlap graph [4]. In this sense, GRASP can be used as a gene-centric assembly tool that reconstructs homologous protein sequences of the reference. It can also be used as a homolog-search tool to recruit individual homologous reads, by using the assembled contigs as templates. Benchmark data showed that GRASP had improved the recall rate of existing homolog search tools from ~ 40% to ~ 60% without loss of precision [4]. Recently developed homolog search tools, such as RAPSearch [5] and DIAMOND [6] focused on improving computational efficiency, and have lower performances in recall rate. Hence, although many efficient homolog search tools are available, GRASP remains the one with the highest recall rate and overall performance.

However, due to de novo assembly performed in GRASP, it is much slower than traditional read-based homolog search tools such as BLAST. Furthermore, GRASP also requires a large amount of memory to store a suffix array data structure used for assisting with the assembly step. Hence, GRASP had only been applied to relatively simple and small metagenomic data sets such as those generated from the human oral environment. To extend GRASP’s practical utility, we developed a new simultaneous alignment and assembly algorithm called GRASPx [7]. GRASPx improves the speed of GRASP by ~30X without compromising the original performance of GRASP. GRASPx also uses a similar amount of memory as GRASP. Despite significant performance improvement, GRASPx requires more time and space than other homolog search tools.

In this article, we present a completely redesigned simultaneous alignment and assembly algorithm called GRASP2 to further improve the computational and space efficiency upon GRASPx. We benchmark GRASP2 against a set of homolog search algorithms including GRASPx, BLAST, PSI-BLAST, and FASTM. The benchmark results show that GRASP2 is 8-fold faster than GRASPx and uses 8-fold less memory for both of its indexing and assembly/search phases. GRASP2 has the same high performance compared to GRASPx; and GRASP2 has significantly outperformed the other homolog search tools. These results suggest our novel algorithm can effectively reduce the running time and space requirement of the simultaneous alignment and assembly algorithms. The resulting software GRASP2 has great application potential for its high performance and significantly improved computational efficiency.

## Methods

### The original GRASPx algorithmic framework

We first summarize the GRASPx’s algorithmic framework to identify its limitations that have been further improved here. GRASPx contains two main stages, i.e., the indexing stage for building a suffix array from the entire read set to facilitate efficient overlap detection, and the assembly/search stage that searches the conceptual overlap graph for paths that correspond to homologs of the reference protein.

In its indexing stage, GRASPx uses extension links to represent the read overlap information [7]. An extension link exists between a source read and a target read, if the suffix of the source and the prefix of the target share a common *l*-mer. The extension links are built by a linear scan of the suffix array constructed from the entire read set to identify intervals that share a common prefix of length longer than *l* (set to 10 amino acids for both GRASPx and GRASP2 as default). It implies that all reads whose suffixes are included in such intervals share a common *l*-mer, thus allowing us to build the corresponding extension links. There are four limitations of the extension link representation. First, GRASPx requires the construction of a suffix array from the entire read set, which may not fit into memory when the dataset is huge. Second, GRASPx linearly scans the constructed suffix array, which is typically implemented as a single-threaded program to effectively use the pre-fetch feature of modern computer architectures. Therefore, GRASPx’s indexing phase has low multithreading scalability. Third, the extension link is added between two reads as long as they share a common *l*-mer, ignoring whether the two reads indeed overlap. This formulation is equivalent to the construction of assembly *de Bruijn* graph [8], which is not read coherent and may include false overlap information between reads [9]. Finally, the extension links that correspond to a single, unbranched path in assembly graphs are redundant. These extension links should be contracted to reduce the number of extensions and save memory space.

GRASPx’s assembly/search phase involves iterative assembly and alignment similar to GRASP. GRASPx first identifies a seed read, which shares a common *k*-mer (set to 6 for both GRASPx and GRASP2 as default) with the reference protein. The seed read is used to initialize an *open contig*, which is defined as a contig that can be potentially extended. In the assembly step, GRASPx extends the open contig by following the extension links incident from the last read of the open contig. (GRASPx also symmetrically extend the open contig from the C-terminal to N-terminal.) In the alignment step, GRASPx aligns the reference protein with the newly extended open contig. Extended open contigs remain open if the resulting alignment score with the reference protein is higher than a threshold; otherwise, they are *closed* to terminate further extensions. The algorithm terminates when all contigs are closed, and outputs all paths that result in significant alignment scores with the reference protein. This approach, however, does not effectively utilize the pre-fetch feature of modern computer architecture. The alignment step often keeps a two-dimensional dynamic programming table in cache, and it flushes the information pre-fetched from the extension link table away from the cache/main memory. Extension links thus need to be reloaded for the next assembly step. Similarly, the assembly step loads in the extension links and flushes out the dynamic programming table, requiring the algorithm to reload the dynamic programming table for the next alignment step.

The redesigned GRASP2 algorithm improves GRASPx in the following aspects. To improve the memory consumption of GRASPx’s indexing phase, GRASP2 uses a more memory-efficient data structure, i.e. Burrows-Wheeler Transformation (BWT) and FM-index [10] to replace the original suffix array data structure for read indexing. GRASP2’s indexing phase also strictly require read overlap rather than *k*-mer sharing during the read overlap phase. GRASP2 generates the assembly string graph during its index phase, which is read-coherent and contains no false positive edges compared to *de Bruijn* graphs. To improve GRASPx’s assembly/search phase, GRASP2 decomposes the assembly and alignment steps into two independent components to allow more efficient pre-fetch. GRASP2’s assembly/search phase only requires the string graph as input, which correspond to contracted extension links; therefore, it is also more memory efficient than GRASPx.

### GRASP2: assembly string graph creation

GRASP2 aims at generating the complete string graph from the read set. Although existing assemblers are capable of generating the assembly graphs as intermediate results, those graphs are often extensively processed or pruned (e.g., bubble removal, tip trimming, repeat copy number prediction). The processed assembly graphs often suffer from information loss, especially SNPs or small indels from low-abundance genomes. Direct use of the processed graph as output by existing assemblers will render low homolog search recall rate. Hence, GRASP2 generates its own SNP-aware string graph directly from raw sequencing reads.

**Fig. 1:**
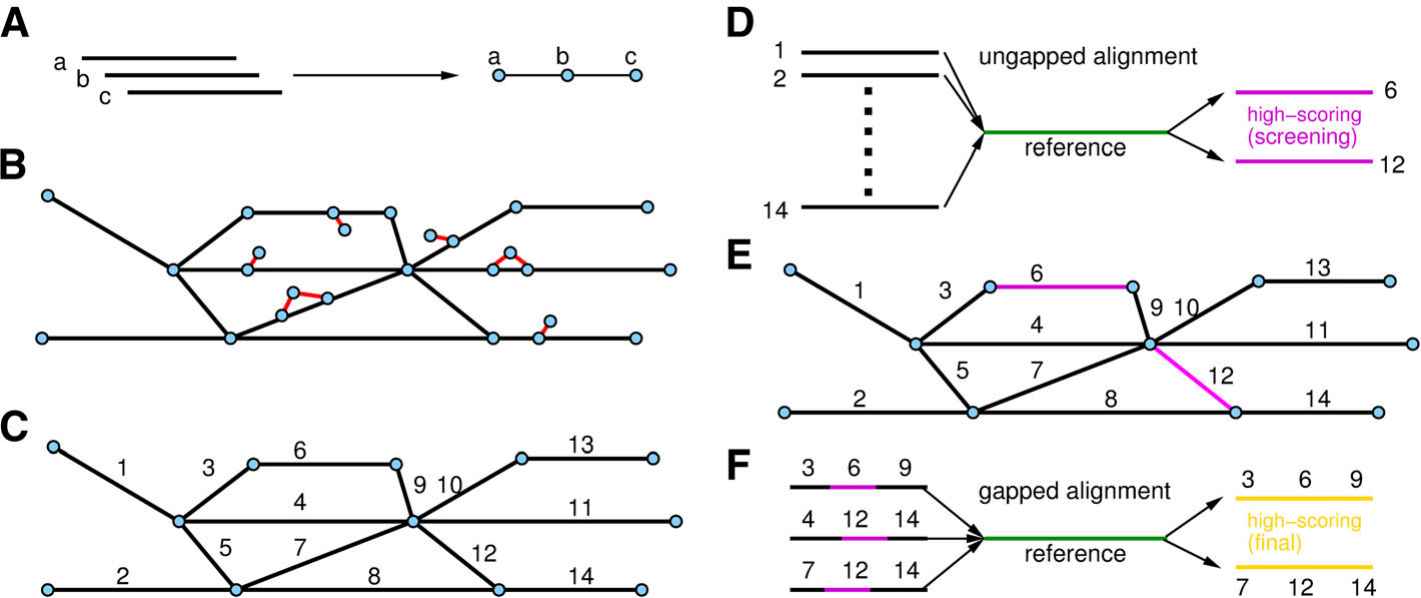
Overview of the GRASP2 algorithm. **a** The reads are overlapped to construct an assembly string graph. **b** Bubbles and tips are identified and subsequently removed from the assembly graph. **c** Unbranched paths are collapsed into single edges. Each collapsed edge is considered as a unitig and receives an arbitrary label. **d** Ungapped alignment is performed between the reference protein sequence and each of the unipath. **e** High-scoring unipaths are treated as anchors to initialize gapped alignments. **f** Candidate paths are generated based on the identified anchor unipaths. Gapped alignment is performed between the reference protein sequence against the candidate paths to recruit homolog paths

String graph generation has been introduced in [9]. The computational bottleneck of this algorithm is overlap detection. A linear-time overlap detection algorithm, using BWT and the FM-index, has been previously proposed [11]. GRASP2 adopts a modified version of this algorithm to detect read overlaps, and subsequently construct the string graph (Fig. 1a). To consider large data sets, GRASP2 partitions the raw read set into smaller blocks and constructs BWT for each block. The size of each data block is a tunable parameter, which allows the user to control the memory consumption of the indexing stage. For each read, GRASP2 then searches for all BWT blocks to identify the read’s overlapping neighbors. Note that the final set of overlapping neighbors of the read is not a simple union of the ones detected from the BWT blocks, because *contained reads* may fail to be identified if they are partitioned into different BWT blocks. However, simple union of overlapping neighbors is computationally efficient; therefore, GRASP2 adopts this approach initially and subsequently rectifies the resulting errors after the graph is constructed.

Specifically, let *r* be the current read being processed, and *r*_*A*_ be an overlapping read identified form data block *A* and *r*_*B*_ be one identified from data block *B*. If *r*_*A*_ is not contained in *r*_*B*_ (as a substring) nor vice versa, *r*_*A*_ and *r*_*B*_ can be directly connected with *r* because they correspond to different paths in the assembly graph. On the other hand, assume (without loss of generality) that *r*_*A*_ is contained in *r*_*B*_, *r* should only be overlapped with *r*_*B*_. Two scenarios are possible. First, *r*_*A*_ and *r*_*B*_ may subsequently both overlap with another read *r*^′^. Because we only consider overlaps with maximum lengths [9], overlap between *r*_*A*_ and *r*^′^ also implies overlap between *r*_*B*_ and *r*^′^, and vice versa. In this case, two identical paths exist, i.e., (*r*, *r*_*A*_, *r*^′^) and (*r*, *r*_*B*_, *r*^′^), which correspond to a bubble in the assembly graph. Because the two paths are identical, one needs to be removed to reduce redundancy of the assembly graph. To remove the bubble, for each node *v* in the graph, we extend it in both directions up to a certain depth (tunable parameter). Then, for each node *u* that have been visited during the extension, we also extend it in both directions to check whether there exists another alternative *v* − *u* path. If yes, the sequences of the two *v* − *u* paths are then aligned. One of the *v* − *u* path is eliminated from the assembly graph only if the corresponding two sequences are identical; in this case, our algorithm can retain polymorphism information derived from low-abundant genomes.

In the second scenario, *r*_*B*_ overlaps with *r*^′^, but because *r*_*A*_ is contained in and is shorter than *r*_*B*_, *r*_*A*_ does not overlap with *r*^′^ for a length that larger than the required overlap length *l*. In this case, the path (*r*, *r*_*A*_) is terminated and corresponds to a tip in the assembly graph. Clearly, the tip (*r*, *r*_*A*_) is redundant since the corresponding sequence information is already contained in the path (*r*, *r*_*B*_, *r*^′^). We first identify tips from the assembly graph as terminating nodes (or beginning nodes by symmetry) whose corresponding path contains only two nodes, with one being the tip itself and the other connecting to other longer paths (contain at least three nodes or more). Note that orphan two-node paths, i.e. those without at least one node connecting to a longer path, are retained. Similar to the bubble removal step, the sequence of the tip is also compared with all neighbors of its connecting node to the major path; the tip node is removed only when the tip sequence is found to be contained in other paths. We summarize the bubble removal and tip trimming steps as Fig. 1b (nodes connected by red edges). After bubble and tip removal, the remaining graph is simplified by collapsing nodes from unbranched paths [9] (Fig. 1c). The sequences represented by the condensed edges are correct and are guaranteed to be present in any assembly of the read set; the sequences are hereafter referred to as *unitigs*.

### GRASP2: novel algorithm for simultaneous alignment and assembly

The assembly/search stage of GRASP2 aims at searching the string graph to identify paths (as a concatenation of multiple unitigs) whose corresponding sequences are homologous to the reference protein. These paths are called *homologous paths*. The algorithmic design of GRASP2 aims at uncoupling the string graph access and alignment to reduce page faults and achieve speedup over GRASPx. The first step of GRASP2’s assembly/search algorithm is to identify candidate unitigs (Fig. 1c, unitigs are labeled arbitrarily to ease algorithm presentation) that are likely contained in homologous paths. Similar to BLAST, GRASP2 performs ungapped extension (or alignment) based on seed pairs identified between the reference protein and the unitigs (Fig. 1d, unitigs 1 through 14). Because all *k*-mers have already been identified during the previous indexing stage, the ungapped alignment step is very efficient. Unitigs that receive ungapped alignment scores higher than a threshold are retained as *anchors* for candidate homologous path identification (Fig. 1e, purple edges; we assume that the ungapped alignments between the reference and the unitigs 6 as well as 12 result in high ungapped score).

The second step of GRASP2’s assembly/search stage is to identify all candidate homologous paths, *defined as paths that contain at least one anchor*. GRASP2 first sorts the anchors in descending order based on their ungapped alignment scores (in Fig. 1e, we assume the score for unitig 6 is higher than the score for unitig 12). Then, based on the highest-ordered anchor in the list, GRASP2 performs depth-first search (DFS) towards both the N-terminal and C-terminal to exhaustively identify all candidate homolog paths. The DFS is initialized based on the position of the ungapped alignment, and its depth is determined to make the constructed candidate paths match the length of the reference protein sequence. During graph traversal, the DFS may reach unitigs that are also in the anchor list; these unitigs are subsequently removed from the anchor list to avoid redundant traversal of the same regions in the string graph. The DFS may also reach unitigs that are previously included in other candidate homolog paths; further extensions from the visited unitigs are also prohibited for a similar reason. For example, in Fig. 1, the DFS based on the anchor unitig 6 will result in a candidate path (3, 6, 9), and the DFS based on the anchor unitig 12 will result in candidate path (9,12,14), (4,12,14), and (7,12,14). However, because the unitig 9 is already included in the candidate path (3, 6, 9), the candidate path (9,12,14) is considered as redundant because it also contains the unitig 9 (Fig. 1f, only three candidate path remain). Because the anchor unitigs are sorted based on the ungapped alignment score at the beginning (i.e. the anchor unitig 6 has a higher ungapped alignment than 12), anchors that have higher ungapped sequence similarity also have higher priority when recruiting its neighbor unitigs to form candidate paths.

The third step is to perform gapped alignment between the reference protein and the identified candidate homologous paths to generate accurate alignment scores and detailed alignments (Fig. 1f). Because each candidate homologous path may contain multiple anchor unitigs, there could exist multiple seed pairs identified between the reference protein and the candidate homolog path. Hence, instead of extending the alignment from each seed pair, GRASP2 directly performs Smith-Waterman local alignment [12] between the reference protein and the candidate homologous paths. In addition, because the candidate homologous paths are identified to match the length of the reference protein, the highest-score match is expected to come from the diagonal of the alignment matrix. This intuition allows us to apply a banded version of the Smith-Waterman algorithm for speedup. We note that the Smith-Waterman algorithm is more sensitive than extending the alignment from each seed pair because the later approach fixes the alignment between the seeds, and partly contributes to the higher recall rate of GRASP2. The candidate homologous paths that receive high local alignment scores are output as homologous contigs of the reference protein.

The last step of GRASP2 is similar to that of GRASPx, which uses the assembled homologous contigs as templates to recruit the original short-peptide reads. This step allows GRASP2 to be used as a homolog search tool in additional to a gene-centric assembly tool. This mapping step adopts traditional read-mapping algorithms that utilize BWT, for example those implemented in BWA [13] and Bowtie [14], with the only difference that GRASP2 operates on amino acid alphabet rather than nucleotide alphabet.

We note that GRASP2’s assembly/search algorithm effectively uncouples string graph access and alignment. The first ungapped alignment step requires no information from the string graph and only scans through the unitig database. The second candidate homologous path identification step operates on the string graph, but performs no alignment and therefore does not require access to the read sequences nor the dynamic programming tables. The third step (Smith-Waterman banded alignment) and the fourth step (read mapping) require no string graph information and directly work on sequence alignments. Therefore, GRASP2 more effectively reduces page faults.

### Benchmark data set creation

We constructed a simulated marine metagenomic data set using fully sequenced bacterial genomes. We selected 20 marine microbial genomes included strains of *Candidatus pelagibacter*, *Prochlorococcus marinus*, *Synechococcus, Flavobacteriales*, *Nitrosococcus oceani*, *Vibrio*, *Photobacterium*, *Erythrobacter*, *Alteromonas*, *Roseobacter* and *Shewanella*. The detailed genome accession IDs and their relative abundances can be found from the Supplementary Table 1 of [3]. These selected fully sequenced genomes are referred to as *core genomes*. We used WGSIM [15] to generate simulated reads from the core genomes at 10X coverage, with a read length of 100 bp and an error rate of 1% (for Illumina technology). We then used FragGeneScan [2] to call short peptides from the simulated reads, which resulted in 6,273,043 short peptide reads in our final simulated data set.

We then generated the ground-truth homology annotation for each read in the simulated data set as follows. Given a reference protein, we searched the protein against the core genomes using TBLASTN [16] and collected identified genomics regions with an E-values cutoff of 10^−3^. We call these regions *homolog regions*. Then, we treated all reads that were derived from the homolog regions as *homologous reads*; and those that were not as *non-homologous reads*. For reads that lie on the boundaries of the homologous regions (referred to as *boundary reads*), we considered them as neither homolog nor non-homologous reads. This is because the boundary reads only contains partial homolog sequences. Treating the boundary reads as homologous reads is unfair for the BLAST suite and FASTM, because these methods may perform poorly in identifying extremely short homolog sequences; while treating the boundary reads as non-homologous reads is unfair for GRASP2 and GRASPx, because these reads indeed contain partial homolog sequences that can be identified based on the read-overlapping information that is available from the reconstructed string graph. We constructed two sets of references protein sequences. The first set contains 198 marker protein sequences identified from *Dehalococcoides sp. CBDB1* by the Amphora2 database [17] (we refer to this reference protein set as the *marker gene reference set*). The second set contains all annotated protein sequences (1442 in total) in the *Dehalococcoides sp. CBDB1* genome (we refer to this reference protein set as the *whole genome reference set*). Note that *D. sp. CBDB1* is not a core genome for generating the simulated dataset; the average sequence similarity between *D. sp. CBDB1*. and the selected marine microbial genomes is 76.9% (with min 75.1% and max 78.7%).

## Results

### De novo assembly fails to recruit the majority of homolog reads

We benchmarked the performance for the strategy of using de novo nucleotide assembly tools to assist homolog detection. Given the above set of simulated nucleotide reads, SOAPdenovo2 [18] and SPAdes [19] were used to assemble the simulated reads using an in-house server equipped with two Intel Xeon X5687 CPUs and 96GB of RAM. Both assemblers were run with default parameters. Then, each protein sequence within the marker gene reference set was searched against the contigs to identify homolog regions using TBLASTN under *E*-value cutoffs ranging from 10^6^ to 10^−10^. In a contig interval is aligned with multiple queries, the interval is assigned to the query that resulted in the most significant *E*-value. Then, to assign the individual reads to queries, we mapped the reads against the assembled contigs using BWA [13] with default parameters. Individual reads aligned within (or have > 60% of their overall lengths overlap with) homolog contig intervals were then assigned to the corresponding queries.

We defined TP (true positive) as the homologous reads that are identified, FP (false positive) as the non-homologous reads that are identified, and FN (false negative) as the homologous reads that are not identified. Correspondingly, we computed the recall and precision rates using the below formula:

$$ recall=\frac{TP}{TP+ FN}; precision=\frac{TP}{TP+ FN}. $$

We observed that the homolog-search performances of this strategy is low in both recall and precision rates. Specifically, the best *F*-measure of the de novo assembly strategy using SOAPdenovo2 is achieved at the *E*-value cutoff of 10^−4^, with a recall rate of 3.90%, a precision rate of 6.39%, and a *F*-measure of 4.84%. Similarly, for using SPAdes, the best *F*-measure is achieved at the *E*-value cutoff of 10^−4^, with a recall rate of 3.93%, a precision rate of 6.41%, and a *F*-measure of 4.87%. The reason for such low performances is due to de novo nucleotide assembly being a very challenging problem, impacted a great deal by nucleotide polymorphisms. Reliable assemblies also depend on the availability of high-quality and high-coverage sequencing data. For our simulated data set, although the target coverage was set to 10X at the beginning, WGSIM considers sequencing coverage bias during the simulation, rendering uneven coverage for different genomic regions. Low-coverage regions are difficult to assemble, leading to fragmentary assemblies for both assemblers. Specifically, SOAPdenovo2 has an N50 of 43,607 bp and SPAdes has an N50 of 83,315 bp. The total length of all 20 reference genomes is 73,392,806 bp, while the total length of the contigs generated by SOAPdenovo2 is 4,570,376 bp (~ 6.23% genome coverage) and that generated by SPAdes is 4,704,253 bp (~ 6.40% genome coverage). The low reference-genome coverage rate leads to low recall rates, as the majority of the genomes are not assembled. In addition, the assemblies are also found to be chimeric. We counted the number of different genomes whose reads are involved in each single contigs. For SOAPdenovo2, each contig involves reads from 8.45 genomes on average, and for SPAdes each contig involves reads from 7.80 genomes on average. The high chimera rate leads to low precision, as each contig interval could potentially involve false positive reads that were originally derived from other reference genomes. In consequence, de novo assembly may not be an ideal strategy to annotate individual reads based on homolog search.

### Benchmark of GRASP2 with other homolog search tools

We benchmarked the performance of GRASP2 with GRASPx [7], BLASTP [16], PSI-BLAST [16], and FASTM [20]. The version of BLAST in use is NCBI version 2.3.0. The version of FASTM in use is version 36. We did not include GRASP into this benchmark since it is too slow, and its performance is similar to that of GRASPx [7]. GRASP2, GRASPx, and BLASTP were executed with the default set of parameters, and PSI-BLAST was run with three iterations. For FASTM, we use the “-BP62” option to specify the use of the BLOSUM62 scoring matrix, which is also used by the other software tested here. An *E*-value cutoff range from 10^6^ to 10^−10^ were applied to all programs to measure their performances under different cutoff stringencies. All experiments were performed on an in-house server equipped with two Intel Xeon X5687 CPUs and 96GB of RAM. We then used the recall and precision measured on different *E*-value cutoffs to generate the ROC (Receiver Operating Characteristics) curves for each software.

**Fig. 2:**
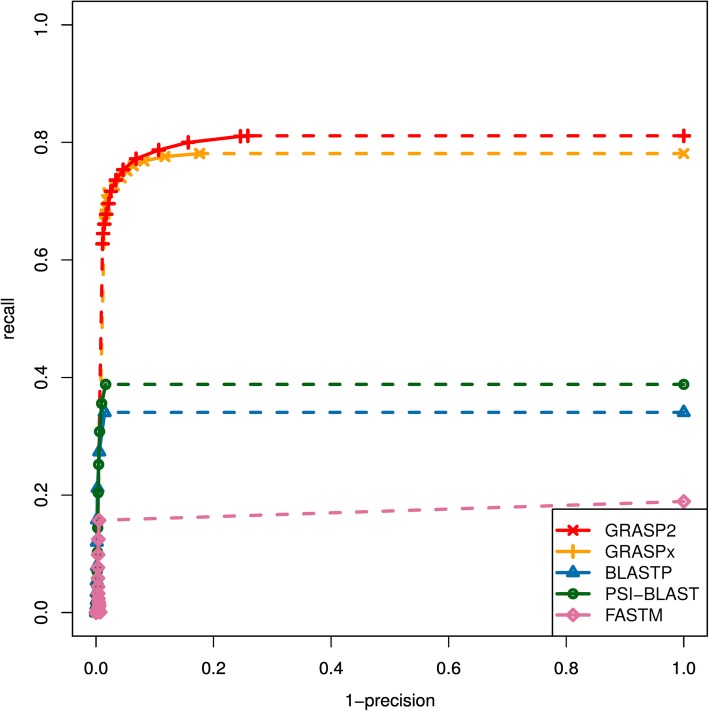
The Receiver Operating Characteristic (ROC) curves for the performances of GRASP2, GRASPx, BLASTP, PSI-BLAST, and FASTM on the marker gene reference set. GRASP2 and GRASPx have the highest performance among all programs that have been benchmarked. The dotted lines correspond to extrapolated performances

**Fig. 3:**
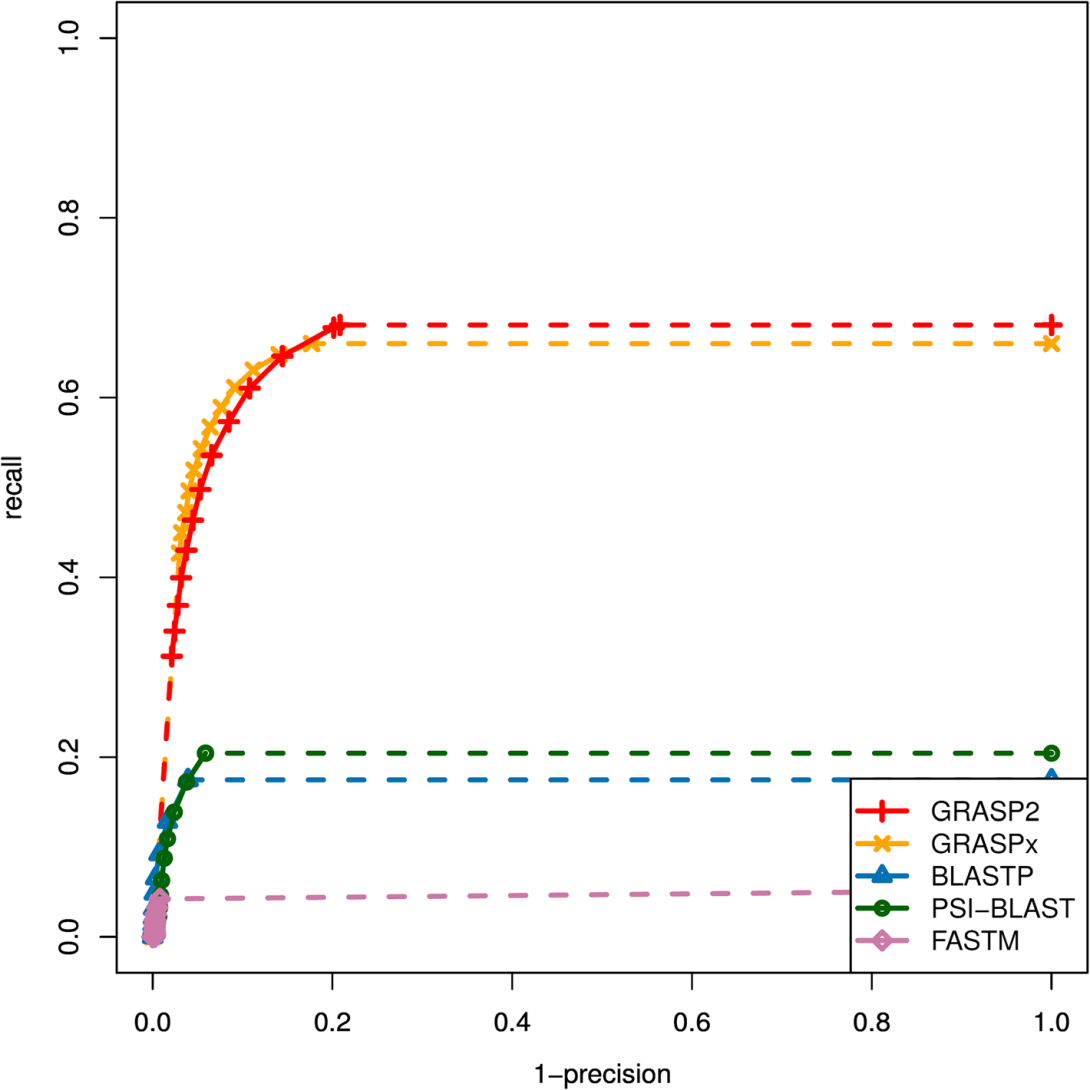
The Receiver Operating Characteristic (ROC) curves for the performances of GRASP2, GRASPx, BLASTP, PSI-BLAST, and FASTM on the whole genome reference set. GRASP2 and GRASPx have the highest performance among all programs that have been benchmarked. The dotted lines correspond to extrapolated performances

The performance benchmark results are summarized in Fig. 2 (for marker gene reference set) and Fig. 3 (for whole genome reference set). While searching with the marker gene reference set (Fig. 2), GRASP2 and GRASPx have the highest performances among all programs that have been tested. At a high-precision level (~ 90% precision rate, *E*-value cutoff 10^−1^), GRASP2 and GRASPx have respectively 78.7 and 76.8% recall rate, compared to 21.2% of PSI-BLAST (the best among the rest of the tested programs). Similarly, while searching with the whole genome reference set (Fig. 3), GRASP2 and GRASPx also have the highest performances. At a high-precision level (~ 90% precision rate, *E*-value cutoff 10^−1^), the recall rate of GRASP2, GRASPx, and PSI-BLAST is 60.4, 63.1, and 9.2%, respectively. For both reference sets, the recall rate of BLAST, PSI-BLAST, and FASTM cannot be further improved even with extremely high *E*-value cutoff (10^6^), suggesting that no seed pairs may be identified between the reference protein and those missed homologous reads. GRASP2 and GRASPx were able to detect these reads because these reads may be overlapped with other reads that contain one or more seeds. Overall, GRASP2 and GRASPx have similar performances, which suggest that the completely redesigned GRASP2 algorithm is capable of retaining the original high performance with memory reduction and speedup.

**Fig. 4:**
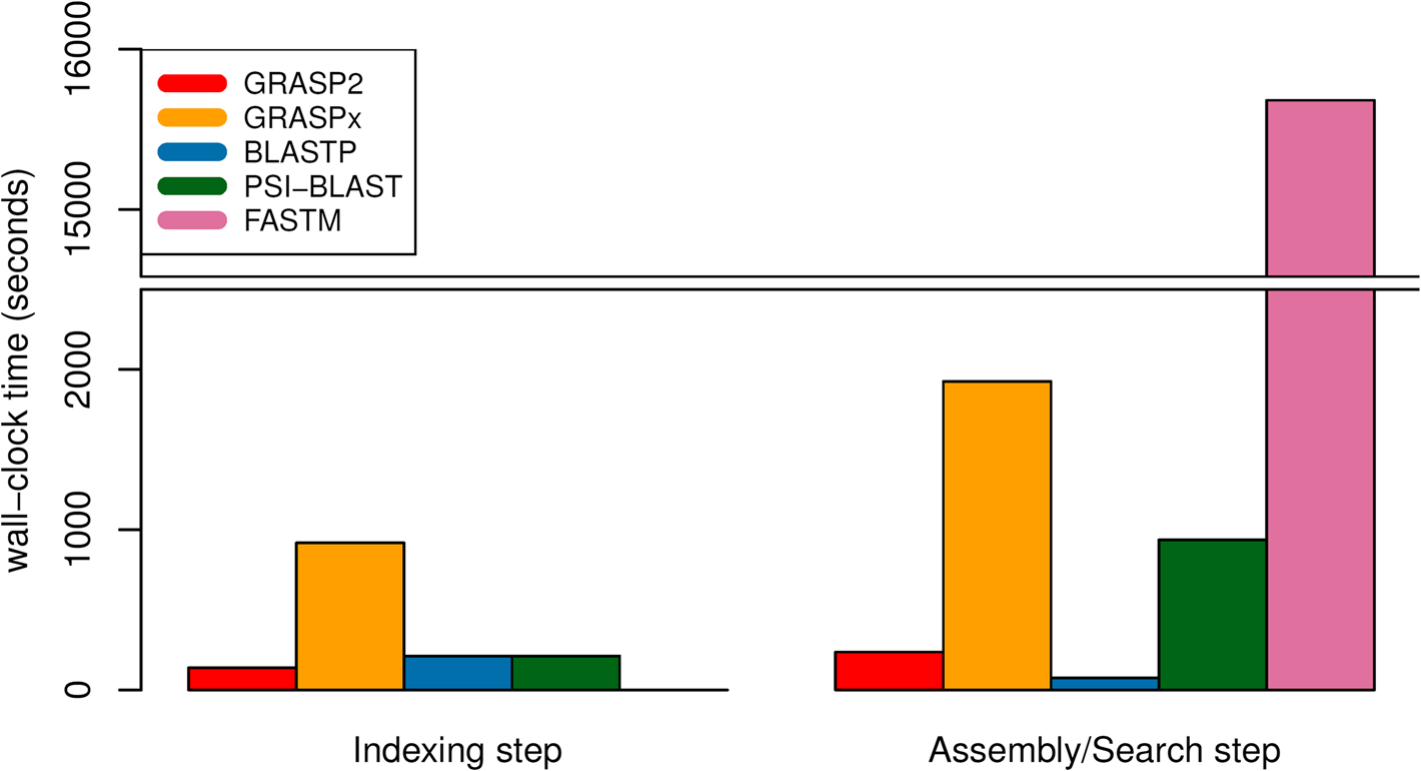
Running time comparison (for both the indexing and assembly/search steps) among GRASP2, GRASPx, BLASTP, PSI-BLAST, and FASTM on the marker gene reference set

**Fig. 5:**
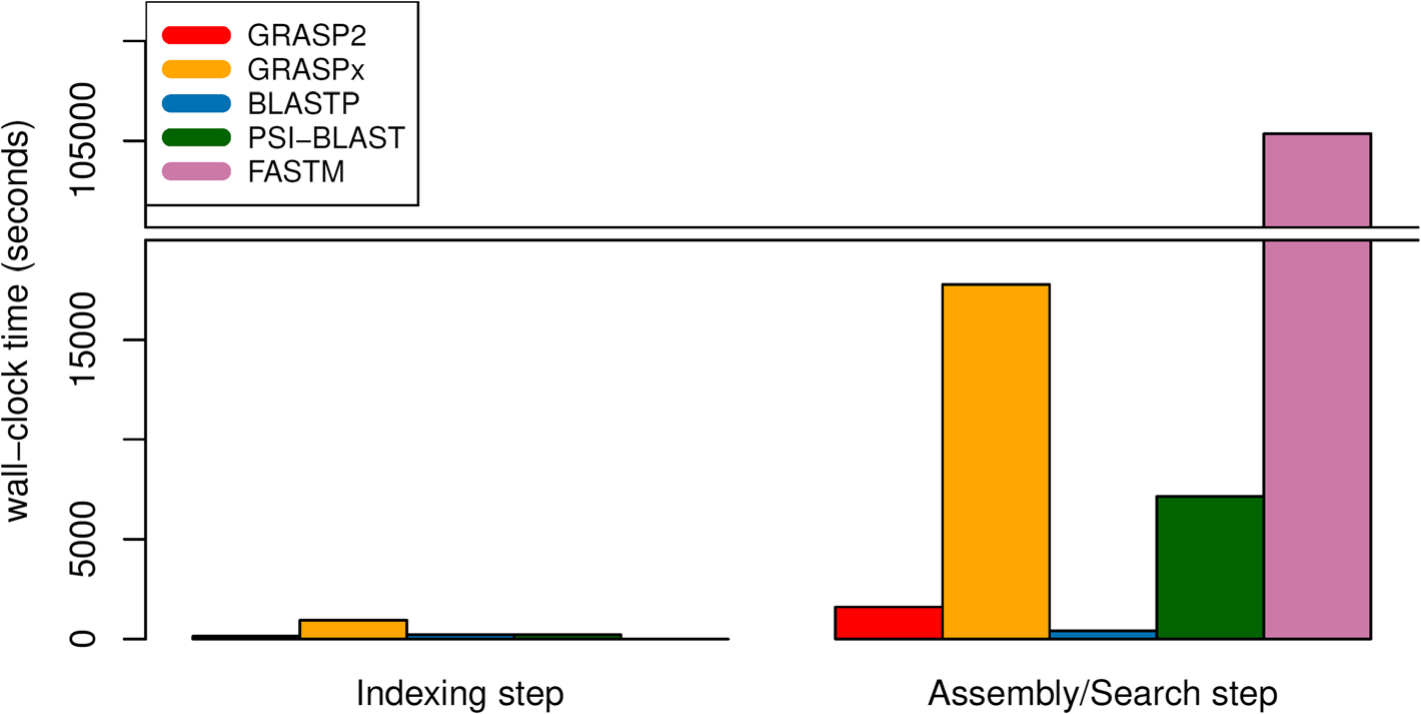
Running time comparison (for both the indexing and assembly/search steps) among GRASP2, GRASPx, BLASTP, PSI-BLAST, and FASTM on the whole genome reference set

The wall-clock run times of the software is summarized in Fig. 4 (for marker gene reference set) and Fig. 5 (for whole genome reference set). For the indexing step (that is independent of the reference set), GRASP2 is the fastest software (excluding FASTM that does not perform indexing), and is 6.6-fold faster than GRASPx (139 s vs. 918 s) and 1.5-fold faster than the BLAST suite (139 s vs. 213 s). Note that GRASP2’s indexing step was run with 8 threads. GRASP2’s indexing step includes overlap detection among all input reads, which can be easily parallelized to reduce the actual running time. The other software, including GRASPx, BLAST, and PSI-BLAST, cannot make use of multiple threads for indexing (the corresponding indexing programs “graspx-build” and “makeblastdb” do not contain options to allow executions in multi-threaded mode) and thus were run under a single-threaded mode.

For the assembly/search step, all programs were run with 8 threads. When searching the marker gene reference set (Fig. 4), GRASP2 is 8.1-fold faster than GRASPx (238 s vs. 1924 s) and 3.9-fold faster than PSI-BLAST (238 s vs. 936 s). GRASP2 is also 3.2-fold slower than BLASTP (238 s vs. 75 s). FASTM has significantly longer running time (15,681 s) compared to the other software, potentially because it does not employ an indexing step to speed up the actual alignment. A similar pattern is also observed while searching with the whole genome reference set (Fig. 5). GRASP2 is 11.2-fold faster than GRASPx (1587 s vs. 17,800 s) and 4.5-fold faster than PSI-BLAST (1587 s vs. 7148 s). GRASP2 is also 3.8-fold slower than BLASTP (1587 s vs. 413 s). However, we also note that GRASP2 assigns the search of each reference protein to a dedicated thread, and thus has high scaling efficiency when run with more computing resources. The extra running time required by GRASP2 can easily be ameliorated by introducing more computing nodes.

**Fig. 6:**
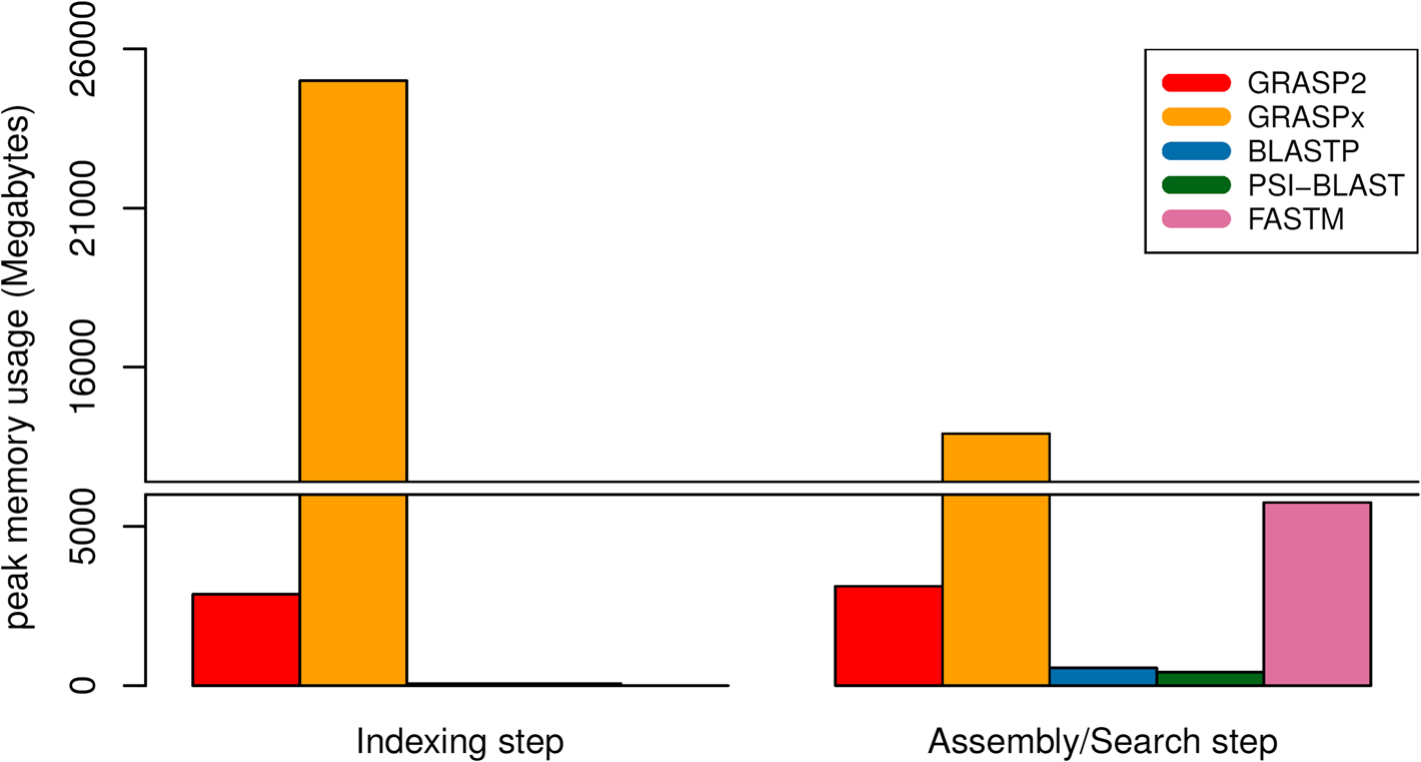
Peak memory consumption comparison (for both the indexing and assembly search steps) of GRASP2, GRASPx, BLAST, PSI-BLAST, and FASTM on the marker gene reference set

**Fig. 7:**
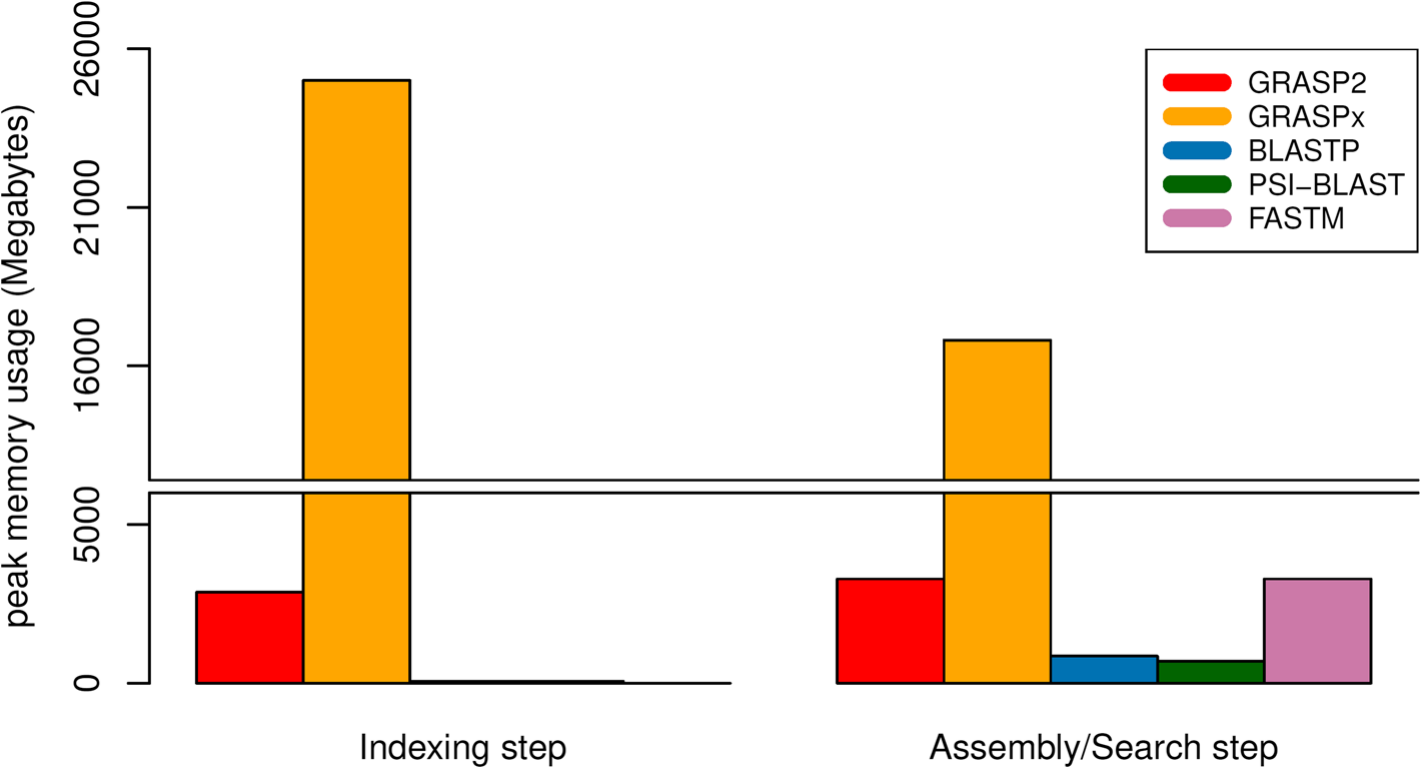
Peak memory consumption comparison (for both the indexing and assembly search steps) of GRASP2, GRASPx, BLAST, PSI-BLAST, and FASTM on the whole genome reference set

Finally, the peak memory consumption of the software is summarized in Fig. 6 (for marker gene reference set) and Fig. 7 (for whole genome reference set). The memory consumption for all software primarily comes from holding the target dataset in memory, and remains relatively invariant even with a reference set with a much larger size. In both cases (Fig. 6 and Fig. 7), GRASP2 and GRASPx have higher memory requirement, because they perform de novo assembly of the reads and need to construct and/or retain the assembly graph in memory. When searching the marker gene reference set (Fig. 6), GRASP2 has significantly reduced the memory consumption of both GRASPx’s indexing step (8.9-fold, 2.8GB vs. 25.0GB) and GRASPx’s assembly/search step (4.5-fold, 3.1GB vs. 13.9GB). Overall, the peak memory consumption for the entire GRASPx pipeline including both the indexing and the assembly/search stages has been reduced by 8.1-fold (3.1GB vs. 25.0GB). Similar memory consumption can also be observed when searching the whole genome reference set (Fig. 7). In practice, GRASP2 requires ~3G RAM to index and assemble/search ~ 6 million reads (as in the benchmark). Such a lower memory requirement makes possible the analysis of metagenomic data sets with medium or medium-high complexity (e.g., human gut metagenomics data that contains ~ 100–200 million reads can be analyzed using ~500GB-1TB RAM).

## Discussions

In this article, we present a novel simultaneous alignment and assembly algorithm called GRASP2. The completely redesigned GRASP2 algorithm makes it 8-fold faster and uses 8-fold less memory than its predecessor GRASPx. GRASP2 is only three times slower than the popular homolog search tool BLAST, but can generate much higher quality results that have 20–40% higher recall rate at the same precision level. The improved computational efficiency and reduced memory consumption, together with its high recall and precision, make GRASP2 a potentially useful tool for metagenomics data analysis.

For future work, we plan to further improve GRASP2’s efficiency using the following strategies. First, GRASP2 employs the reduced protein alphabet that is currently used in RAPSearch [5] for seeding. The seeds correspond to traditional *k*-mers that are consecutive in sequence. We will further explore the use of spaced seeds [21]. The spaced seed strategy has been implemented in DIAMOND [6] to increase its search speed without losing sensitivity. Second, GRASP2 implements thread-level parallelism that performs assembly/search simultaneously on multiple threads. We plan to further explore data-level parallelism using the SIMD (Single Instruction Multiple Data) scheme for speeding up the Smith-Waterman alignment stage. SIMD has been implemented in DIAMOND and HMMER3 [22], and well-implemented SIMD libraries for Smith-Waterman algorithm are available [23]. Finally, we will explore DIAMOND’s strategy of indexing both the reference proteins and the read set (called *double indexing*). The double indexing strategy will further improve the speed of GRASP2 when a large reference protein database (e.g., the NCBI NR database) is used to query a metagenomics data set. We anticipate further running time improvement of GRASP2 when these strategies are implemented and optimized with the existing GRASP2 algorithmic framework.

## Conclusions

The main contribution of this work is the development of the GRASP2 algorithm, which is a computation- and memory-efficient redesign over its predecessor GRASPx. In addition, we demonstrate here the possibility of improving all simultaneous alignment and assembly algorithms using the “filter, traverse, then align” strategy as implemented in GRASP2. For example, this strategy could be directly applied to improve protein-family homolog search with profile-HMM. We anticipate that GRASP2 will be used in more metagenomic sequencing data analysis projects to produce highly sensitive and accurate functional annotation. GRASP2, implemented in C++, is open-source and freely available from http://www.sourceforge.net/projects/grasp2.
